# Patterns of Obesity Development before the Diagnosis of Type 2 Diabetes: The Whitehall II Cohort Study

**DOI:** 10.1371/journal.pmed.1001602

**Published:** 2014-02-11

**Authors:** Dorte Vistisen, Daniel R. Witte, Adam G. Tabák, Christian Herder, Eric J. Brunner, Mika Kivimäki, Kristine Færch

**Affiliations:** 1 Steno Diabetes Center, Gentofte, Denmark; 2 Centre de Recherche Public de la Santé, Strassen, Luxembourg; 3 Department of Epidemiology and Public Health, University College London, London, United Kingdom; 4 1st Department of Medicine, Semmelweis University, Faculty of Medicine, Budapest, Hungary; 5 Institute for Clinical Diabetology, German Diabetes Center, Leibniz Center for Diabetes Research at Heinrich Heine University Düsseldorf, Düsseldorf, Germany; Chinese University of Hong Kong, China

## Abstract

**Background:**

Patients with type 2 diabetes vary greatly with respect to degree of obesity at time of diagnosis. To address the heterogeneity of type 2 diabetes, we characterised patterns of change in body mass index (BMI) and other cardiometabolic risk factors before type 2 diabetes diagnosis.

**Methods and Findings:**

We studied 6,705 participants from the Whitehall II study, an observational prospective cohort study of civil servants based in London. White men and women, initially free of diabetes, were followed with 5-yearly clinical examinations from 1991–2009 for a median of 14.1 years (interquartile range [IQR]: 8.7–16.2 years). Type 2 diabetes developed in 645 (1,209 person-examinations) and 6,060 remained free of diabetes during follow-up (14,060 person-examinations). Latent class trajectory analysis of incident diabetes cases was used to identify patterns of pre-disease BMI. Associated trajectories of cardiometabolic risk factors were studied using adjusted mixed-effects models. Three patterns of BMI changes were identified. Most participants belonged to the “stable overweight” group (*n* = 604, 94%) with a relatively constant BMI level within the overweight category throughout follow-up. They experienced slightly worsening of beta cell function and insulin sensitivity from 5 years prior to diagnosis. A small group of “progressive weight gainers” (*n* = 15) exhibited a pattern of consistent weight gain before diagnosis. Linear increases in blood pressure and an exponential increase in insulin resistance a few years before diagnosis accompanied the weight gain. The “persistently obese” (*n* = 26) were severely obese throughout the whole 18 years before diabetes diagnosis. They experienced an initial beta cell compensation followed by loss of beta cell function, whereas insulin sensitivity was relatively stable. Since the generalizability of these findings is limited, the results need confirmation in other study populations.

**Conclusions:**

Three patterns of obesity changes prior to diabetes diagnosis were accompanied by distinct trajectories of insulin resistance and other cardiometabolic risk factors in a white, British population. While these results should be verified independently, the great majority of patients had modest weight gain prior to diagnosis. These results suggest that strategies focusing on small weight reductions for the entire population may be more beneficial than predominantly focusing on weight loss for high-risk individuals.

*Please see later in the article for the Editors' Summary*

## Abstract 2

Examining patterns of change in body mass index (BMI) and other cardiometabolic risk factors in individuals during the years before they were diagnosed with diabetes, Kristine Færch and colleagues report that few of them experienced dramatic BMI changes.

*Please see later in the article for the Editors' Summary*

## Editors' Summary

**Background:**

Worldwide, more than 350 million people have diabetes, a metabolic disorder characterized by high amounts of glucose (sugar) in the blood. Blood sugar levels are normally controlled by insulin, a hormone released by the pancreas after meals (digestion of food produces glucose). In people with type 2 diabetes (the commonest form of diabetes) blood sugar control fails because the fat and muscle cells that normally respond to insulin by removing sugar from the blood become insulin resistant. Type 2 diabetes, which was previously called adult-onset diabetes, can be controlled with diet and exercise, and with drugs that help the pancreas make more insulin or that make cells more sensitive to insulin. Long-term complications, which include an increased risk of heart disease and stroke, reduce the life expectancy of people with diabetes by about 10 years compared to people without diabetes. The number of people with diabetes is expected to increase dramatically over the next decades, coinciding with rising obesity rates in many countries. To better understand diabetes development, to identify people at risk, and to find ways to prevent the disease are urgent public health goals.

**Why Was This Study Done?:**

It is known that people who are overweight or obese have a higher risk of developing diabetes. Because of this association, a common assumption is that people who experienced recent weight gain are more likely to be diagnosed with diabetes. In this prospective cohort study (an investigation that records the baseline characteristics of a group of people and then follows them to see who develops specific conditions), the researchers tested the hypothesis that substantial weight gain precedes a diagnosis of diabetes and explored more generally the patterns of body weight and composition in the years before people develop diabetes. They then examined whether changes in body weight corresponded with changes in other risk factors for diabetes (such as insulin resistance), lipid profiles and blood pressure.

**What Did the Researchers Do and Find?:**

The researchers studied participants from the Whitehall II study, a prospective cohort study initiated in 1985 to investigate the socioeconomic inequalities in disease. Whitehall II enrolled more than 10,000 London-based government employees. Participants underwent regular health checks during which their weight and height were measured, blood tests were done, and they filled out questionnaires for other relevant information. From 1991 onwards, participants were tested every five years for diabetes. The 6,705 participants included in this study were initially free of diabetes, and most of them were followed for at least 14 years. During the follow-up, 645 participants developed diabetes, while 6,060 remained free of the disease.

The researchers used a statistical tool called “latent class trajectory analysis” to study patterns of changes in body mass index (BMI) in the years before people developed diabetes. BMI is a measure of human obesity based on a person's weight and height. Latent class trajectory analysis is an unbiased way to subdivide a number of people into groups that differ based on specified parameters. In this case, the researchers wanted to identify several groups among all the people who eventually developed diabetes each with a distinct pattern of BMI development. Having identified such groups, they also examined how a variety of tests associated with diabetes risk, and risks for heart disease and stroke changed in the identified groups over time.

They identified three different patterns of BMI changes in the 645 participants who developed diabetes. The vast majority (606 individuals, or 94%) belonged to a group they called “stable-overweight.” These people showed no dramatic change in their BMI in the years before they were diagnosed. They were overweight when they first entered the study and gained or lost little weight during the follow-up years. They showed only minor signs of insulin-resistance, starting five years before they developed diabetes. A second, much smaller group of 15 people gained weight consistently in the years before diagnosis. As they were gaining weight, these people also had raises in blood pressure and substantial gains in insulin resistance. The 26 remaining participants who formed the third group were persistently obese for the entire time they participated in the study, in some cases up to 18 years before they were diagnosed with diabetes. They had some signs of insulin resistance in the years before diagnosis, but not the substantial gain often seen as the hallmark of “pre-diabetes.”

**What Do These Findings Mean?:**

These results suggest that diabetes development is a complicated process, and one that differs between individuals who end up with the disease. They call into question the common notion that most people who develop diabetes have recently gained a lot of weight or are obese. A substantial rise in insulin resistance, another established risk factor for diabetes, was only seen in the smallest of the groups, namely the people who gained weight consistently for years before they were diagnosed. When the scientists applied a commonly used predictor of diabetes called the “Framingham diabetes risk score” to their largest “stably overweight” group, they found that these people were not classified as having a particularly high risk, and that their risk scores actually declined in the last five years before their diabetes diagnosis. This suggests that predicting diabetes in this group might be difficult.

The researchers applied their methodology only to this one cohort of white civil servants in England. Before drawing more firm conclusions on the process of diabetes development, it will be important to test whether similar results are seen in other cohorts and among more diverse individuals. If the three groups identified here are found in other cohorts, another question is whether they are as unequal in size as in this example. And if they are, can the large group of stably overweight people be further subdivided in ways that suggest specific mechanisms of disease development? Even without knowing how generalizable the provocative findings of this study are, they should stimulate debate on how to identify people at risk for diabetes and how to prevent the disease or delay its onset.

**Additional Information:**

Please access these Web sites via the online version of this summary at http://dx.doi.org/10.1371/journal.pmed.1001602.

- The US National Diabetes Information Clearinghouse provides information about diabetes for patients, health-care professionals, and the general public, including information on diabetes prevention (in English and Spanish)
- The UK National Health Service Choices website provides information for patients and carers about type 2 diabetes; it includes people's stories about diabetes
- The charity Diabetes UK also provides detailed information about diabetes for patients and carers, including information on healthy lifestyles for people with diabetes, and has a further selection of stories from people with diabetes; the charity Healthtalkonline has interviews with people about their experiences of diabetes
- MedlinePlus provides links to further resources and advice about diabetes (in English and Spanish)
- More information about the Whitehall II study is available

## Introduction

Obesity is a well-established risk factor for type 2 diabetes; however, it is well-known that patients with type 2 diabetes vary greatly with respect to degree of adiposity at time of diagnosis [1]–[3]. Thus, a better understanding of the heterogeneity of diabetes is important for improving disease prevention and treatment. Recent studies have described trajectories in plasma glucose, insulin sensitivity, beta cell function, and subclinical inflammation related to diabetes before the disease is diagnosed [4]–[6]. These population-level growth curves contribute to aetiological and pathophysiological understanding, but may somewhat oversimplify the complex and heterogeneous disease mechanisms responsible for type 2 diabetes. To facilitate stratified, targeted interventions, identification of population subgroups with similar risk factor patterns seems essential. One way of identifying such groups is to use data-driven statistical methods, such as latent class trajectory analysis [7]. This method identifies distinct classes or subgroups of people who are homogeneous with respect to the development of a given risk factor over time, but heterogeneous as compared with other groups. Although latent class trajectory analysis has been widely used in criminology and behavioural research [8],[9], it is new to health research [10],[11] and has only very recently been applied in a study of diabetes patients [12], but not in relation to diabetes aetiology.

In this study, we aimed to identify different patterns of obesity development over a period of 18 years in a population initially free of diabetes. In addition, we examined trajectories of other metabolic risk factors accompanying each pattern of obesity development.

## Methods

### Ethics Statement

The Whitehall II study was reviewed and approved by the University College London Ethics Committee (85/0938). Written informed consent was obtained from each participant at each phase. The study was conducted according to the principles of the Helsinki Declaration.

### Study Participants

This study uses data from the longitudinal Whitehall II cohort of non-industrial British civil servants. In the original study, a total of 10,308 participants (6,896 men and 3,412 women aged 35–55 years) of mainly white ethnicity who worked in London offices of 20 departments were recruited between 1985 and 1988 (phase 1) and followed at eight subsequent phases ∼2.5 years apart. All study phases included a questionnaire, and every second phase (∼5 years apart) also included a clinical health examination (phases 1, 3, 5, 7, and 9). In the Whitehall II cohort 6,057 men and 2,758 women participated at phase 3 (1991–1993); 5,473 men and 2,397 women at phase 5 (1997–1999); 4,893 men and 2,074 women at phase 7 (2002–2004); and 4,759 men and 2,002 women at phase 9 (2007–2009). The Whitehall II study is described in detail elsewhere [13].

Phase 3 (1991–1993) was the first phase when an oral glucose tolerance test (OGTT) was performed. Therefore, we did not use data from phase 1. With the last follow-up being the phase 9 examination in 2008–2009, this study was based on 9,181 (89.1%) white participants. From these, we excluded 830 (9.0%) participants who were lost to follow-up prior to phase 3, 211 (2.3%) participants with prevalent diabetes at phase 3 (51% based on OGTT and 49% based on a diabetes diagnosis outside the study), 776 (8.5%) participants for which diabetes status could not be assessed at any phase in the study, and another 659 (7.2%) with no measurement of body mass index (BMI) throughout the study.

At phases 3, 5, 7, and 9 a standard 2-hour 75 g OGTT was performed in the morning after an overnight fast (≥8 hours of fasting). For a subset of participants, the OGTT was administered in the afternoon after a light fat-free breakfast (≥5 hours of fasting). OGTT measurements for participants with less than 8 hours of fasting were excluded from the analysis. Diabetes was diagnosed by a doctor outside the study (43.1%) or at screening by OGTTs (56.9%). Screen-detected diabetes was ascertained throughout follow-up by OGTTs administered every 5 years and defined according to the OGTT criteria defined by the World Health Organization (WHO) [14].

Thus, the final sample included 6,705 participants (73.0% of the original sample of white ethnicity) with a median follow-up time of 14.1 years (interquartile range [IQR]: 8.7–16.2 years) and 15,269 person-examinations. We identified 645 (9.6%, 1,209 person-examinations) cases of incident diabetes by phase 9.

**Figure 1:**
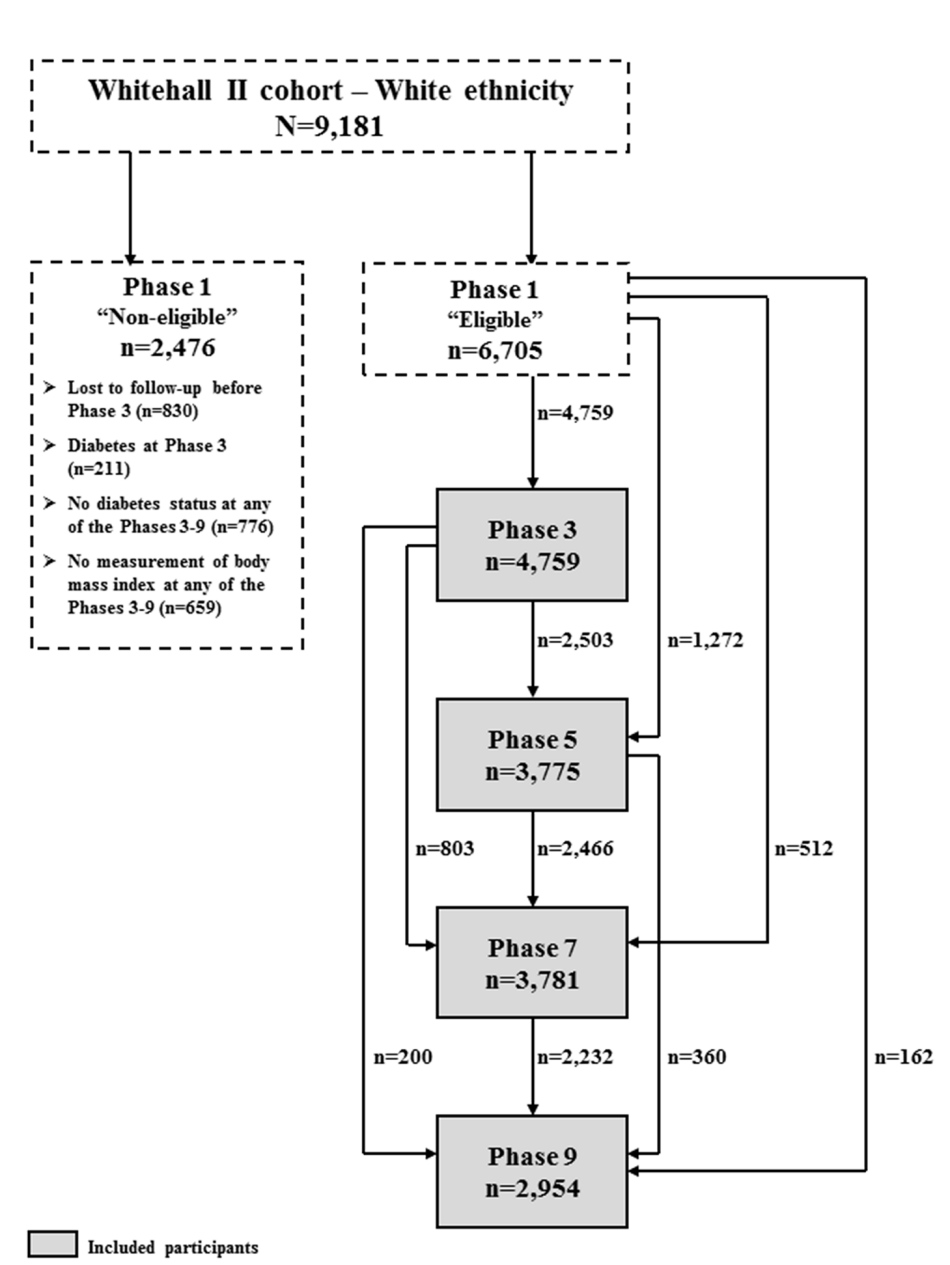
Flow diagram of the participants included at each phase.

A flow diagram of the participants included at each phase is shown in Figure 1.

### Study Procedures and Calculations

Weight, height, and waist circumference were measured according to standard protocols [13] at phases 3, 5, 7, and 9. Systolic and diastolic blood pressure was measured by manual random zero sphygmomanometer at phases 3 and 5 and by OMRON HEM 907 at phases 7 and 9. Data on ethnicity, smoking status, and family history of diabetes were collected by questionnaire at each phase. During all phases of the study, blood samples were handled according to standardized procedures. Blood glucose was measured with the glucose oxidase method [4], and serum insulin was measured with an in-house human insulin radioimmunoassay (phase 3) and a DAKO insulin ELISA kit (DakoCytomation Ltd) in later phases [15]. Serum triglycerides, total cholesterol, and high-density lipoprotein (HDL) cholesterol were measured using automated enzymatic colorimetric methods at all phases. Interleukin 1 receptor antagonist (IL-1Ra) and total adiponectin serum concentrations were measured with the Quantikine ELISA kit (R&D Systems) in a diabetes case-cohort sample [5],[6]. We used Friedewald's formula to calculate low-density lipoprotein (LDL) cholesterol [16]. The homeostasis model assessment was used to estimate β-cell function (HOMA-%B) and insulin resistance (HOMA-IR) [17]. The absolute 8-year risk of developing type 2 diabetes was calculated in all participants using the Framingham diabetes risk score [18]. Moreover, the Framingham cardiovascular disease (CVD) risk score was used to estimate absolute 10-year risk of developing CVD [19].

### Statistical Analysis

For comparison of characteristics between groups we used chi-square test for categorical variables and t-tests for continuous data. A level of significance of 5% was used.

#### Latent class trajectory analysis

The observation period for retrospective trajectories started at the date of diagnosis for those who developed diabetes, and at the last screening or questionnaire phase for those not developing diabetes (year 0). Date of diabetes diagnosis was set to the date of the OGTT for screen-detected diabetes or to the midpoint between dates of first self-reported diabetes and last diabetes-free screening for patients diagnosed by a doctor outside the study.

The latent class trajectory analysis was performed in the population developing diabetes. The model was specified as a linear mixed-effects model with BMI as the dependent variable. The mixed-effects model specification was used to account for the likely correlation of repeated measurements within the same participant. In the analysis we adjusted for age, sex, and study phase in order to identify latent groups with a different BMI development over time not attributed to differences in age, sex, or study phase.

We used a cubic specification for trajectory shape, i.e., both linear, quadratic, and cubic terms for the time before diabetes diagnosis was entered as covariates in the model. We assumed the effects of confounders (age, sex, and study phase) to be the same for all latent classes and looked for latent classes with different regression parameters for time. A linear term for time before diagnosis was used to specify the random effects of the model, i.e., the individual variation around the mean trajectory (of the individual's latent class).

The “*hlme*” function in the “*lcmm*” package in R version 9.15.2 (The R foundation for Statistical Computing) was used to fit the model http://cran.r-project.org/web/packages/lcmm/lcmm.pdf.

*hlme (BMI ∼ t + t^2^ + t^3^ + age + sex + phase*,

*mixture  = ∼ t + t^2^ + t^3^*,

*random  =  ∼ t*,

*subject  =  ‘id’*,

*ng  =  3*,

*data  =  data)*

The number of latent classes needs to be specified a priori. The optimal number of latent classes to describe data is assessed by comparing the fit of models with different number of latent classes. With a prior requirement of at least 2% of the diabetes population in each group to warrant clinical relevance, we used the Bayesian Information Criterion (BIC) to evaluate the models and selected the number of classes for which the model had the lowest BIC. In this study, three latent classes were identified.

Upon the model fit, a posterior probability of membership to each of the identified latent classes was calculated for each participant, who then was assigned exclusively to the class for which the highest probability was obtained. This class-allocation was used in the subsequent analyses of the accompanying cardiometabolic risk factors.

For each identified BMI group, trajectories of the following outcomes were followed backwards in time to the first clinical examination: waist circumference; systolic and diastolic blood pressure; total, HDL and LDL cholesterol; triglycerides; fasting and 2-hour plasma glucose; fasting and 2-hour serum insulin; HOMA-%B and HOMA-IR; the Framingham diabetes and CVD risk scores; adiponectin and IL-1Ra. Prior to analysis, outcomes with highly skewed distributions were log-transformed (fasting and 2-hour serum insulin, HOMA-%B and HOMA-IR, adiponectin and IL-1Ra). For most determinants ≤5% of the values were missing. For plasma glucose and serum insulin the proportions of missing values were slightly higher (7%–18%). The case-cohort sample with measurements of adiponectin and IL-1Ra covered 66% of diabetes cases and 41% of diabetes-free individuals in this study.

We used linear mixed-effects models to estimate trajectories for each group. For those developing diabetes, time dependence was allowed to vary across the BMI groups. Quadratic and cubic terms for time were included in the three BMI groups when significant (two-sided 5% significance level). For individuals not developing diabetes, year 0 is merely a time point in a normal life course, and we therefore fitted the trajectories by linear models. All analyses were adjusted for age, sex, and study phase. Analyses of lipids were further adjusted for lipid-lowering treatment, and analyses of blood pressure were further adjusted for anti-hypertensive treatment. We tested pair-wise differences in growth curves between the BMI groups using the F-test by comparing the curve of contrasts between two BMI subgroups to a straight line with zero slope and through the origin (two-sided 5% significance level). Provided *p*-values therefore relate to curve differences in slope, intercept, or both.

Statistical analyses were performed in R version 9.15.2 (The R Foundation for Statistical Computing) and SAS version 9.2 (SAS Institute).

### Data Sharing

Whitehall II data, protocols, and other metadata are available to the scientific community. Please refer to the Whitehall II data sharing policy on: www.ucl.ac.uk/whitehallII/data_sharing/index.htm.

## Results

### Patterns of Obesity Development

**Figure 2:**
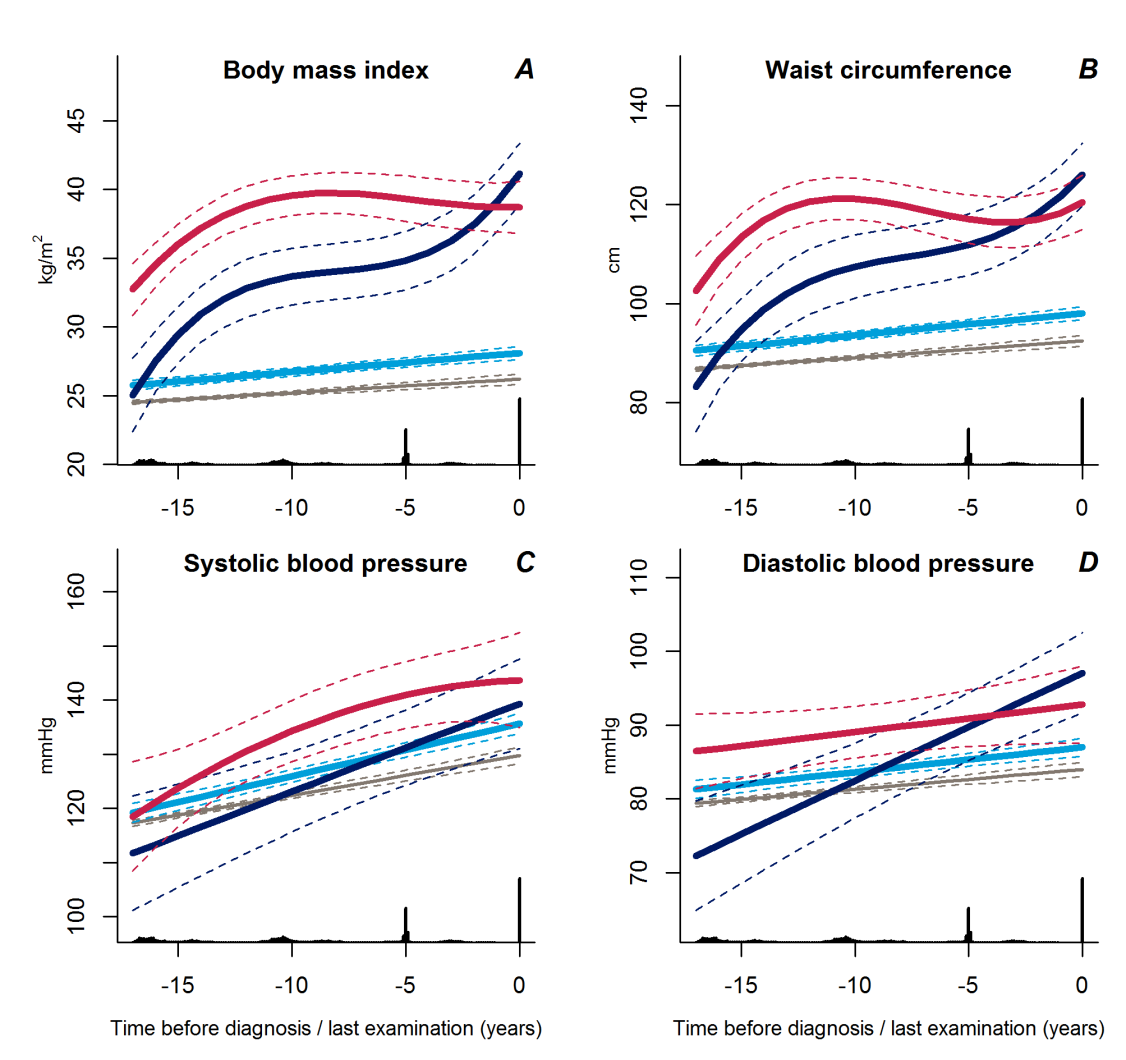
Trajectories for a hypothetical male of 60 years at time 0 of body mass index (A), waist circumference (B), systolic blood pressure (C), and diastolic blood pressure (D) from 18 years before time of diagnosis/last examination. Trajectories for blood pressure represent a person not on anti-hypertensive treatment. Solid lines indicate estimated trajectories for each group and dashed lines are 95% confidence limits. Black bars at the bottom indicate the relative data distribution over the follow-up period. Trajectories of BMI for a hypothetical female of 50 years of age at time of diagnosis are shown in Figure S1. Light blue, stable overweight; dark blue, progressive weight gain; red, persistently obese; grey, diabetes-free population.

We identified three distinct patterns of BMI development prior to diabetes diagnosis (Figure 2A). The latent class group, representing the majority of individuals who developed diabetes, was characterised by an average BMI in the overweight range during the entire follow-up (*n* = 604). This group had an average weight gain of 2.3 BMI units during 18 years of follow-up and was labelled “stable overweight,” because mean BMI was within the overweight category as defined by the WHO (average BMI at time of diagnosis: 28.1 kg/m^2^). Another group had an average BMI in the overweight range more than 15 years before diagnosis, an early weight gain of 8.6 BMI units from 18 to 10 years before diagnosis, then stable BMI in the obese range until 4–5 years before diagnosis. Thereafter, BMI increased to morbid obesity (mean BMI of 41.1 kg/m^2^), totalling a weight gain of 16.1 BMI units during follow-up. This group was termed “progressive weight gainers” (*n* = 15). The third group (*n* = 26), labelled “persistently obese,” was characterised by BMI in the obese range more than 15 years before diagnosis of diabetes (average BMI of 32.7 kg/m^2^), and with persistent obesity until the time of diagnosis. This group had an average weight gain of 6.0 BMI units during the 18 years of follow-up.

The average BMI development in the reference group not developing diabetes was only 1.7 BMI units during the 18 years of follow-up, from 24.5 kg/m^2^ to 26.2 kg/m^2^.

The trajectories of waist circumference (Figure 2B) followed those of BMI for the three BMI trajectory groups with the stable overweight group being significantly different from the progressive weight gainers (*p*<0.001) and the persistently obese (*p*<0.001). The trajectories of waist circumference for the progressive weight gainers and the persistently obese did not differ significantly (*p*≥0.13).

### Trajectories of Blood Pressure and Lipids

**Figure 3:**
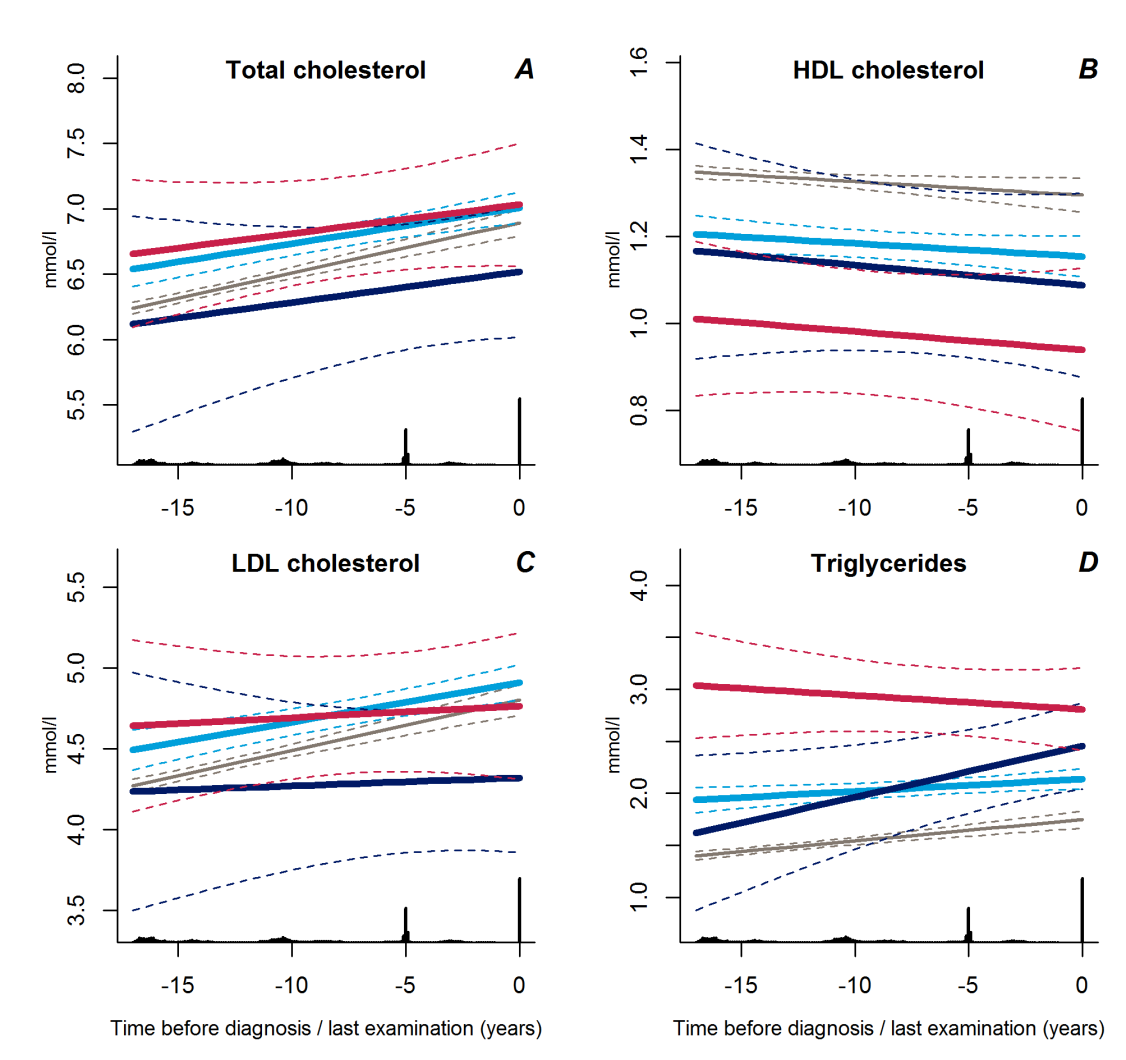
Trajectories for a hypothetical male, not on lipid-lowering treatment, age 60 years at time 0 of total cholesterol (A), HDL cholesterol (B), LDL cholesterol (C), and triglycerides (D) from 18 years before time of diagnosis/last examination. Solid lines indicate estimated trajectories for each group and dashed lines are 95% confidence limits. Black bars at the bottom indicate the relative data distribution over the follow-up period. Light blue, stable overweight; dark blue, progressive weight gain; red, persistently obese; grey, diabetes-free population.

Trajectories of diastolic blood pressure did not differ between the progressive weight gainers and the persistently obese groups (Figure 2D, *p* = 0.18). Individuals with stable overweight exhibited near-normal diastolic blood pressure during follow-up (Figure 2D, *p*<0.001 versus progressive weight gainers, *p* = 0.04 versus persistently obese), although systolic blood pressure did not differ significantly between the groups (Figure 2C, *p*≥0.17). Plasma lipid levels showed a stable pattern towards diabetes diagnosis in the stable overweight group (Figure 3A–3D). LDL cholesterol levels were lower among the progressive weight gainers than among the group of stable overweight (Figure 3C, *p* = 0.010). The progressive weight gainers did not differ from the other groups with respect to other blood lipids (Figure 3, *p*≥0.07 for all). HDL cholesterol was significantly lower during follow-up in the persistently obese compared with the stable overweight group (Figure 3B, *p* = 0.03), whereas triglyceride levels were higher (Figure 3D, *p* = 0.003).

### Trajectories of Insulin and Glucose Metabolism

**Figure 4:**
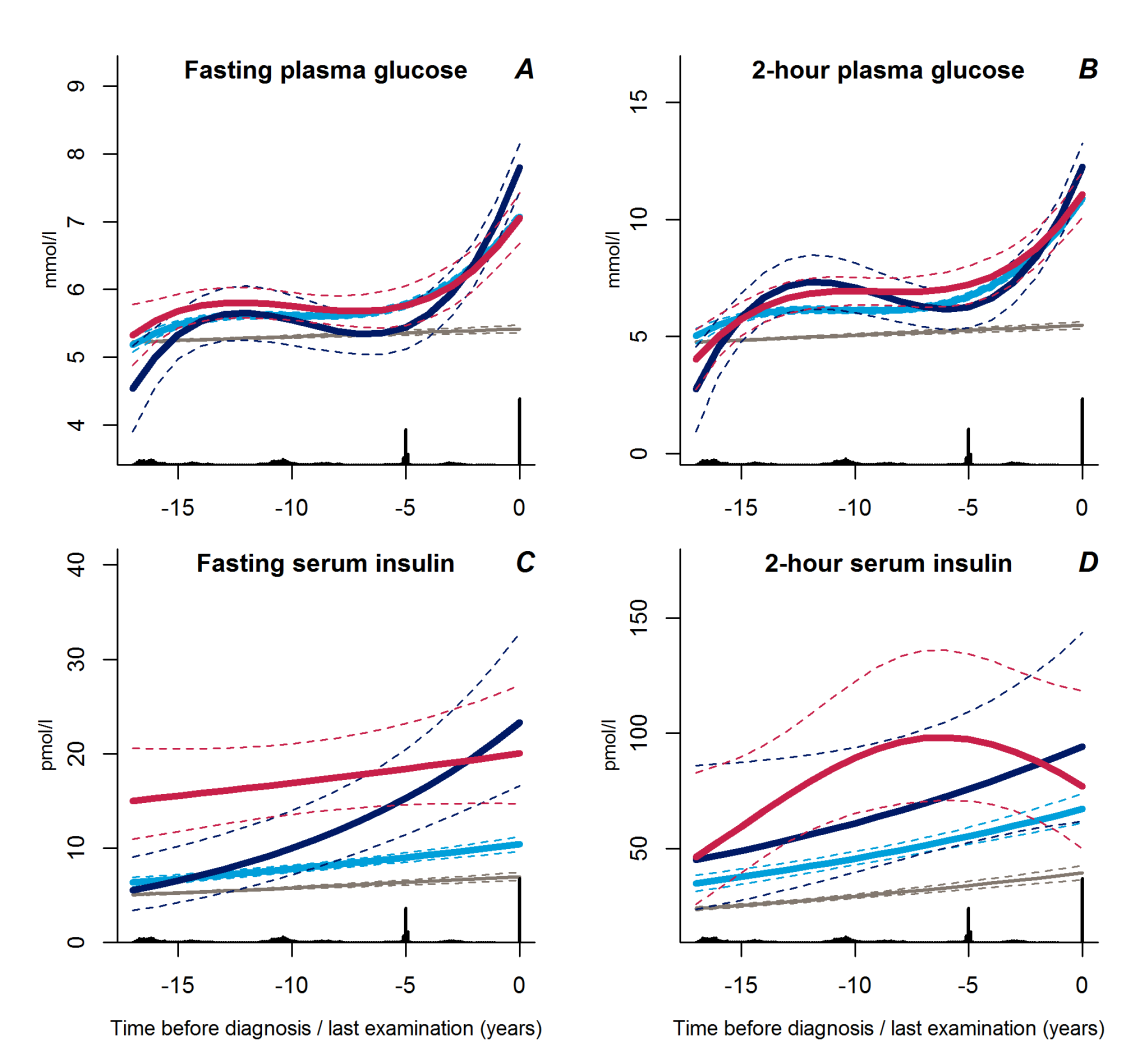
Trajectories for a hypothetical male of 60 years at time 0 of fasting plasma glucose (A), 2-hour plasma glucose (B), fasting serum insulin (C), and 2-hour serum insulin (D) from 18 years before time of diagnosis/last examination. Solid lines indicate estimated trajectories for each group and dashed lines are 95% confidence limits. Black bars at the bottom indicate the relative data distribution over the follow-up period. Light blue, stable overweight; dark blue, progressive weight gain; red, persistently obese; grey, diabetes-free population.

Trajectories of fasting and 2-hour plasma glucose concentrations were similar in all three groups up to a few years before diagnosis at which time point the progressive weight gainers experienced a steep increase (Figure 4A and 4B, *p*<0.02 for all).

Fasting serum insulin concentrations increased exponentially in the group of progressive weight gainers (Figure 4C, *p*≤0.001 versus stable overweight). In contrast, fasting serum insulin concentrations were near-normal in the stable overweight group (Figure 4C, *p*<0.001 versus both other groups). No significant differences in trajectories of 2-hour serum insulin concentration were observed between groups (Figure 4D, *p*≥0.13 for all).

**Figure 5:**
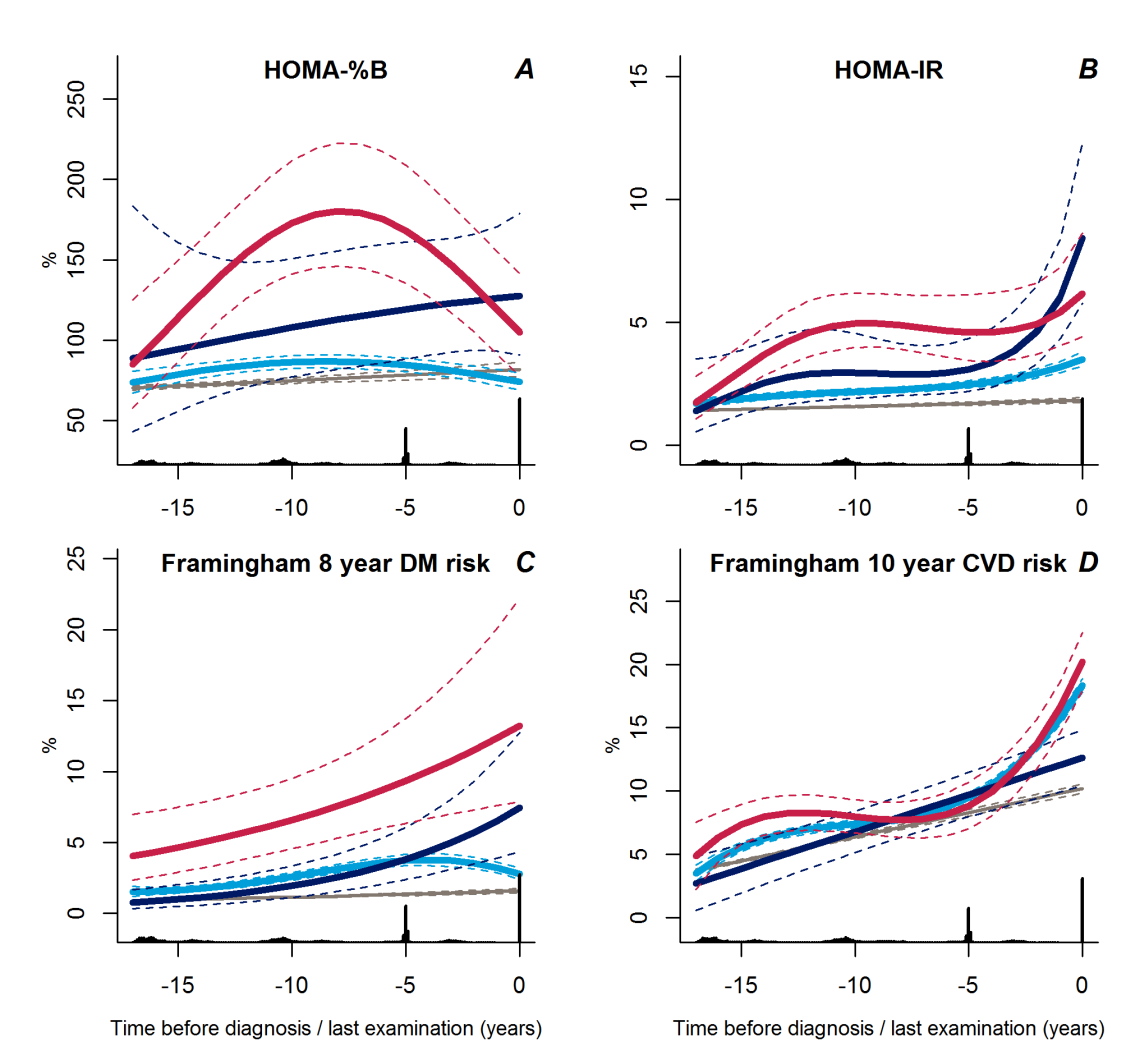
Trajectories for a hypothetical male of 60 years at time 0 of HOMA-%B (A), HOMA-IR (B), Framingham 8-year diabetes risk (C), and Framingham 10-year CVD risk (D) from 18 years before time of diagnosis/last examination. Solid lines indicate estimated trajectories for each group and dashed lines are 95% confidence limits. Black bars at the bottom indicate the relative data distribution over the follow-up period. Light blue, stable overweight; dark blue, progressive weight gain; red, persistently obese; grey, diabetes-free population.

Of interest, the change in fasting serum insulin in the progressive weight gainers was not reflected in the measure of beta cell function, which was stable during follow-up, but at a higher level as compared with the stable overweight group (Figure 5A, *p*<0.001). The shape of beta cell function seemed different in the persistently obese than in the other groups with a classic pattern of beta cell compensation reaching maximum 8 years before diagnosis; however, this difference did not reach statistical significance (*p*≥0.12).

In general, the trajectories of insulin resistance followed those of BMI. The stable overweight group had lower levels of insulin resistance during follow-up than either of the other groups (Figure 5B, *p*≤0.007 for both). Insulin resistance increased rapidly during the last 2–3 years prior to diagnosis in the progressive weight gainers compared with the stable overweight group (Figure 5B, *p*<0.001). In the persistently obese group, insulin resistance increased rapidly more than 10 years before diagnosis, became stable until a few years before diagnosis, and then increased again (Figure 5B, *p* = 0.007 versus stable overweight).

### Trajectories of Estimated Diabetes and CVD Risk

Interestingly, the calculated 8-year diabetes risk declined towards diabetes diagnosis in the large stable overweight group (Figure 5C, *p*<0.001 versus both other groups), whereas calculated diabetes risk was higher in the persistently obese group (Figure 5C, *p*<0.001 versus stable overweight group). Despite the low estimated diabetes risk, the calculated 10-year risk of CVD increased rapidly during the last years prior to diagnosis in the stable overweight and persistently obese groups as compared with the group of progressive weight gainers who experienced a linear increase in calculated CVD risk during follow-up (Figure 5D, *p*<0.001 versus both groups).

### Trajectories of Adiponectin and IL-1Ra

**Figure 6:**
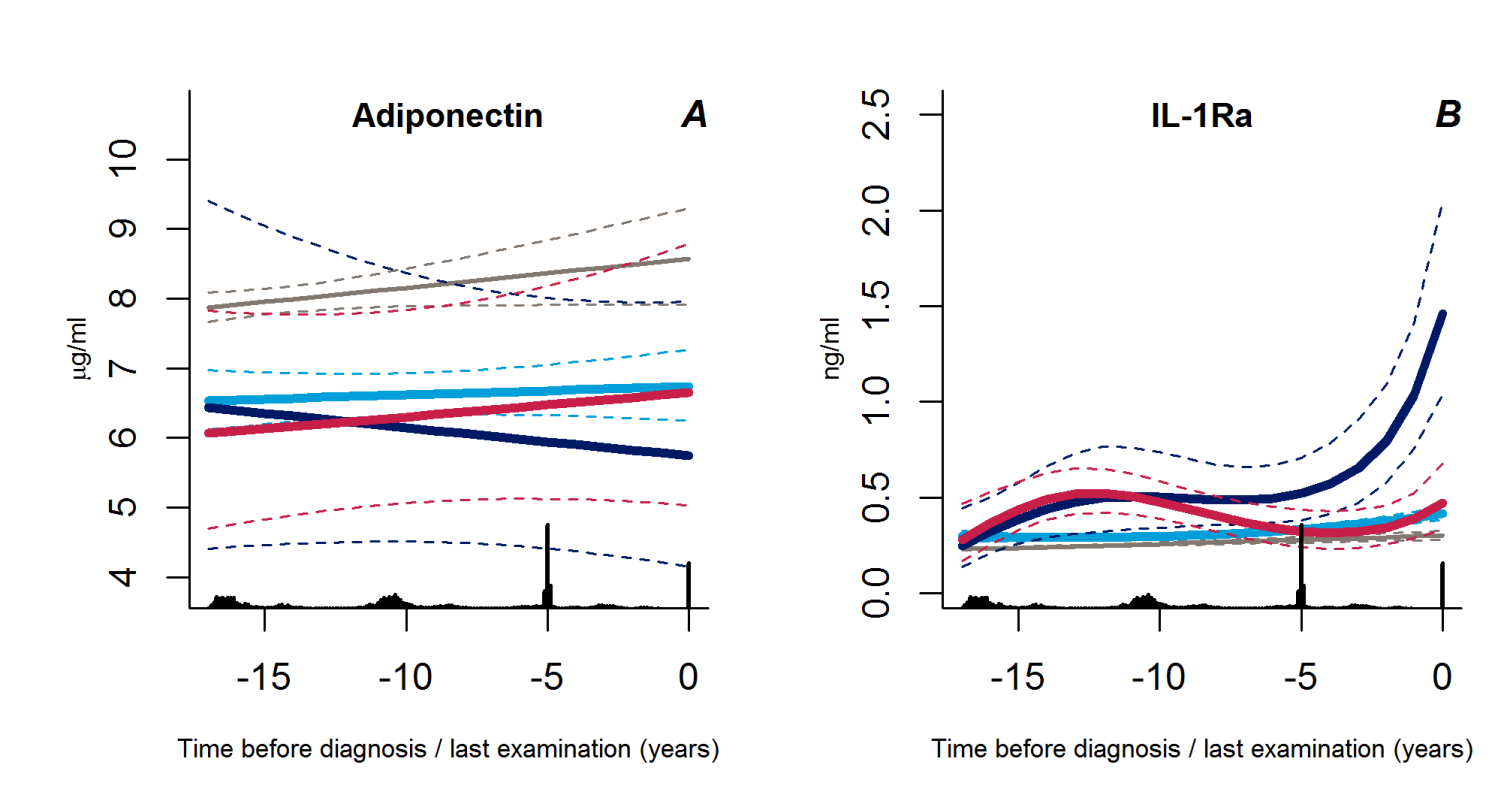
Trajectories for a hypothetical male of 60 years at time 0 of adiponectin (A) and IL-1Ra (B) from 18 years before time of diagnosis/last examination. Solid lines indicate estimated trajectories and dashed lines are 95% confidence limits. Black bars at the bottom indicate the relative data distribution over the follow-up period. Light blue, stable overweight; dark blue, progressive weight gain; red, persistently obese; grey, diabetes-free population.

We found no differences in adiponectin trajectories between the groups (*p*>0.31), but the levels of IL-1Ra increased exponentially in the group of progressive weight gainers as compared with the other groups (p<0.002 for both) (Figure 6A and 6B).

### Other Characteristics

Among those developing diabetes, a significantly higher proportion of the persistently obese than the stable overweight individuals were diagnosed with diabetes by their own general practitioner (*p* = 0.008) (Table 1). The proportion of women was also higher in the persistently obese group than in the stable overweight or diabetes-free groups (*p*<0.02). Furthermore, individuals in the stable overweight and progressive weight gain groups were more likely to have a family history of diabetes compared with the diabetes-free population (*p*<0.04) (Table 1).

**Table 1:** **Characteristics of study participants at time of diagnosis for the three diabetes groups or at the last clinical examination for the diabetes-free group.**

Characteristics	Individuals Developing Diabetes in the Study Stratified by Latent Class BMI Groups			Individuals Free of Diabetes in the Study
	Stablen Overweight (*n* = 604)	Progressive Weight Gain (*n* = 15)	Persistently Obese (*n* = 26)	Diabetes-Free (*n* = 6,060)
Men (%)	74.5 (70.8–77.9)	53.3 (26.6–78.7)	50.0 (29.9–70.1)^a^	71.8 (70.6–72.9)^b^
Ever smoker (%)	56.8 (52.7–60.8)	66.7 (38.4–88.2)	57.7 (36.9–76.6)	51.6 (50.3–52.8)^a^
Family history of diabetes (%)	19.9 (16.8–23.3)	26.7 (7.8–55.1)	11.5 (2.4–30.2)	9.8 (9.1–10.6)^a^^,^ c
Diagnosed by GP outside study (%)	42.2 (38.2–46.3)	33.3 (11.8–61.6)	69.2 (48.2–85.7)^a^^,^ c	–
Ever on anti-hypertensive treatment (%)	27.6 (24.1–31.4)	20.0 (4.3–48.1)	38.5 (20.2–59.4)	22.9 (21.9–24.0)^a^
Ever on lipid lowering treatment (%)	14.4 (11.7–17.5)	13.3 (1.7–40.5)	15.4 (4.4–34.9)	15.7 (14.8–16.7)
Age at diagnosis/last exam. (years)	60.6 (7.8)	61.1 (8.2)	57.9 (7.7)	60.3 (7.9)

Data are percentages (95% CI) or means (SD). Test of difference in characteristics between groups: chi-square test for categorical variables and t-test for continuous data, respectively.

Significantly different.

a Significantly different from stable overweight.

b Significantly different from persistently obese.

c Significantly different from progressive weight gain.

The characteristics of the participants' first clinical examination in the study are shown in Table 2. Since the time span from the first clinical examination to the diagnosis of diabetes differed between the groups, the data should not be compared directly.

**Table 2:** **Characteristics of study participants at their first clinical examination.**

Characteristics	Individuals Developing Diabetes in the Study Stratified by Latent Class BMI Groups			Individuals Free of Diabetes in the Study
	Stable Overweight (*n* = 604)	Progressive Weight Gain (*n* = 15)	Persistently Obese (*n* = 26)	Diabetes-Free (*n* = 6,060)
Time before diabetes diagnosis/last examination (years)	10.3 (5.6–12.7)	6.6 (5.0–13.0)	10.9 (9.7–15.2)	14.3 (9.0–16.2)
Men (%)	74.5 (70.8–77.9)	53.3 (26.6–78.7)	50 (29.9–70.1)^a^	71.8 (70.6–72.9)^b^
Smoker (%)	14.9 (12.2–18.0)	13.3 (1.7–40.5)	23.1 (9.0–43.6)	12.0 (11.2–12.9)^a^
Family history of diabetes (%)	19.9 (16.8–23.3)	26.7 (7.8–55.1)	11.5 (2.4–30.2)	9.8 (9.1–10.6)^a^^,^ c
Antihypertensive treatment (%)	14.6 (11.9–17.6)	6.7 (0.2–31.9)	23.1 (9.0–43.6)	8.5 (7.9–9.3)^a^^,^ b
Lipid lowering treatment (%)	4.5 (3.0–6.4)	0	0	2.4 (2.0–2.8)^a^
Age (years)	52.7 (7.0)	52.9 (7.3)	50.0 (5.9)	51.6 (7.4)^a^
Fasting plasma glucose (mmol/l)	5.7 (1.1)	5.6 (1.5)	5.6 (0.7)	5.2 (0.5)^a^^,^ b ^,^ c
2-hour plasma glucose (mmol/l)	6.6 (2.3)	7.1 (3.0)	6.6 (2.1)	5.2 (1.4)^a^^,^ b ^,^ c
BMI (kg/m^2^)	27 (3.8)	34.2 (8.7)^a^	38.2 (5.0)^a^^,^ c	25.3 (3.6)^a^^,^ b ^,^ c
Waist circumference (cm)	92.3 (11.8)	99.6 (18.0)^a^	112.0 (10.9)^a^^,^ c	86.8 (11.7)^a^^,^ b ^,^ c
Height (cm)	172.4 (9.1)	167.4 (9.4)^a^	166.5 (10.4)^a^	172.9 (9.1)^b^^,^ c
Total cholesterol (mmol/l)	6.6 (1.2)	6 (1.2)	6.8 (1.0)^c^	6.3 (1.2)^a^^,^ b
HDL cholesterol (mmol/l)	1.3 (0.4)	1.3 (0.2)	1.2 (0.4)	1.5 (0.4)^a^^,^ b
LDL cholesterol (mmol/l)	4.4 (1)	3.9 (1.1)	4.5 (0.9)	4.2 (1.1)^a^
Triglycerides (mmol/l)	1.9 (1.3)	1.8 (0.6)	2.8 (2.1)^a^^,^ c	1.3 (0.9)^a^^,^ b
Systolic blood pressure (mmHg)	125.4 (15.5)	122.7 (22.3)	128.0 (14.9)	120.6 (14.3)^a^^,^ b
Diastolic blood pressure (mmHg)	81.4 (10.3)	80.1 (14.1)	85.5 (9.7)^a^	78.1 (9.9)^a^^,^ b
Fasting serum insulin (pmol/l)	8.5 (5.4–13.3)	12.5 (7.5–31.5)^a^	15.9 (10.2–20.7)^a^	5.8 (3.9–8.7)^a^^,^ b ^,^ c
2-hour serum insulin (pmol/l)	52.0 (29.4–86.4)	56.5 (46.9–96.5)	84.3 (44.8–140.7)^a^	30.6 (17.6–50.8)^a^^,^ b ^,^ c
HOMA-IR	2.2 (1.4–3.5)	3.2 (1.9–7.3)^a^	3.8 (2.5–5.2)^a^	1.5 (1.0–2.2)^a^^,^ b ^,^ c
HOMA-%B	86.4 (60.5–131.5)	132.8 (123.7–153.3)^a^	155.5 (109.4–214.8)^a^	77.3 (56–112)^a^^,^ b ^,^ c
Adiponectin (mg/ml)	7.4 (5.3–10.8)	7.4 (7.0–9.7)	7.9 (5.0–12.6)	9.0 (6.6–12.8)^a^
CRP (µmol/L)	1.3 (0.7–2.6)	1.8 (1.4–3.2)	4.4 (2.4–6.6)^a^	0.8 (0.4–1.7)^a^^,^ b ^,^ c
IL-6 (µmol/l)	1.6 (1.2–2.3)	1.6 (1.1–3)	2.5 (2.1–3.8)^a^	1.3 (1.0–2.0)^a^^,^ b

Data are percentages (95% CI), means (SD) or medians (IQR). Test of difference in characteristics between groups: chi-square test for categorical variables and t-test for continuous data, respectively.

a Significantly different from stable overweight.

b Significantly different from persistently obese.

c Significantly different from progressive weight gain.

### Time Effects and Posterior Probability Memberships

Estimated beta coefficients (SE) for the time effects are shown in Table 3. In the stable overweight group, a cubic specification of time (t^3^) was only included in the models for fasting and 2-hour plasma glucose, HOMA-IR, and estimated diabetes and CVD risk. The trajectories for diastolic blood pressure, cholesterol, and triglyceride were linear for all groups.

**Table 3:** **Fixed effects of time dependence for the different outcome variables in the multilevel models of change before the diagnosis of diabetes for the diabetes groups and before the end of follow-up for the diabetes-free population.**

	Stable Overweight			Progressive Weight Gainers			Persistently Obese			Diabetes-Free
	t	t^2^	t^3^	t	t^2^	t^3^	t	t^2^	t^3^	t
BMI	0.095 (0.015)	–	–	2.285 (0.314)	0.268 (0.054)	0.011 (0.002)	0.016 (0.269)	0.060 (0.042)	0.005 (0.002)	0.056 (0.012)
Waist circumference	0.284 (0.048)	–	–	4.832 (1.238)	0.553 (0.211)	0.024 (0.009)	2.558 (1.074)	0.537 (0.169)	0.026 (0.007)	0.173 (0.035)
Systolic BP	0.462 (0.081)	–	–	1.158 (0.383)	–	–	−0.280 (0.970)	−0.072 (0.059)	–	0.248 (0.049)
Diastolic BP	0.236 (0.055)	–	–	1.379 (0.266)	–	–	0.265 (0.228)	–	–	0.183 (0.032)
Total cholesterol	0.001 (0.006)	–	–	−0.010 (0.030)	–	–	−0.016 (0.025)	–	–	0.022 (0.003)
HDL cholesterol	−0.006 (0.002)	–	–	−0.008 (0.008)	–	–	−0.007 (0.007)	–	–	−0.006 (0.001)
LDL cholesterol	0.002 (0.006)	–	–	−0.023 (0.027)	–	–	−0.026 (0.024)	–	–	0.019 (0.003)
Triglycerides	0.014 (0.005)	–	–	0.051 (0.024)	–	–	−0.013 (0.019)	–	–	0.023 (0.003)
Fasting glucose	0.444 (0.018)	0.045 (0.003)	0.001 (0.000)	0.898 (0.099)	0.105 (0.017)	0.004 (0.001)	0.471 (0.093)	0.053 (0.014)	0.002 (0.001)	0.006 (0.002)
2-hour glucose	1.391 (0.051)	0.139 (0.008)	0.004 (0.000)	2.443 (0.290)	0.314 (0.050)	0.012 (0.002)	1.403 (0.260)	0.162 (0.040)	0.006 (0.002)	0.012 (0.005)
Fasting insulin	0.026 (0.003)	–	–	0.082 (0.017)	–	–	0.014 (0.014)	–	–	0.016 (0.002)
2-hour insulin	0.025 (0.004)	–	–	0.030 (0.022)	–	–	−0.093 (0.053)	–0.006 (0.003)	–	0.017 (0.003)
HOMA-%B	−0.037 (0.008)	−0.002 (0.000)	–	0.011 (0.046)	−0.001 (0.003)	–	−0.138 (0.034)	–0.009 (0.002)	–	0.010 (0.002)
HOMA-IR	0.104 (0.017)	0.009 (0.003)	0.000 (0.000)	0.376 (0.105)	0.044 (0.018)	0.002 (0.001)	0.156 (0.085)	0.026 (0.013)	0.001 (0.001)	0.012 (0.002)
Estimated DM risk	−0.171 (0.033)	−0.024 (0.005)	−0.001 (0.000)	0.124 (0.027)	–	–	0.060 (0.023)	–	–	0.023 (0.003)
Estimated CVD risk	2.575 (0.099)	0.265 (0.016)	0.009 (0.001)	0.279 (0.087)	–	–	3.713 (0.500)	0.417 (0.077)	0.014 (0.003)	0.076 (0.012)
Adiponectin	−0.005 (0.003)	–	–	−0.014 (0.011)	–	–	−0.002 (0.009)	–	–	−0.002 (0.003)
IL-1RA	0.051 (0.008)	0.002 (0.000)	–	0.385 (0.085)	0.044 (0.015)	0.002 (0.001)	0.232 (0.092)	0.040 (0.014)	0.002 (0.001)	0.013 (0.002)

Data are beta-coefficients (SE).

BP, blood pressure; DM, diabetes mellitus.

The mean posterior probability of class membership for individuals was high for each of the classes (75%–96%) (Table 4).

**Table 4:** **Average class probabilities by latent classes.**

Latent Class	Mean of Posterior Probabilities		
	Class 1	Class 2	Class 3
Class 1: stable overweight	0.955	0.027	0.018
Class 2: progressive weight gainers	0.113	0.779	0.108
Class 3: persistently obese	0.151	0.103	0.746

## Discussion

By use of latent class trajectory analysis we identified three distinct patterns of obesity development prior to the diagnosis of type 2 diabetes: (1) a stable overweight group, (2) a group of progressive weight gainers, and (3) a persistently obese group. The patterns of obesity development were accompanied by different trajectories of insulin resistance and other cardiometabolic risk factors, underscoring that type 2 diabetes is a not a single disease entity, but rather a heterogeneous disease with different pathophysiological pathways depending on the level and development of obesity.

In contrast to common belief, the great majority of patients diagnosed with diabetes did not have a substantial weight gain prior to diagnosis (stable overweight group). Their average obesity development was comparable to the reference group not developing diabetes, with a slightly higher initial BMI level of 1.2 kg/m^2^ and ending at a 1.9 unit higher BMI level at time of diagnosis. This group of patients experienced a slightly worsening of beta cell function and insulin sensitivity starting ∼5 years before they were diagnosed with diabetes. A previous study found that leaner patients with type 2 diabetes may have a stronger genetic predisposition than obese patients [20]. Our finding of a higher proportion of individuals with a family history of diabetes among the stable overweight group compared with the diabetes-free group supports this notion. Of particular interest is the finding that the persistently obese group did not have a higher proportion of individuals with a family history of diabetes compared with the diabetes-free group, which may have prevented them from developing diabetes earlier in their life despite their high degree of obesity.

Interestingly, the estimated 8-year diabetes risk from the Framingham diabetes risk score remained relatively low and even decreased during the last 5 years prior to diabetes diagnosis in the stable overweight group. Prediction of diabetes in this group may therefore be difficult using established validated models. The finding of a significantly higher proportion of the persistently obese individuals being diagnosed with diabetes by their own general practitioner as compared with the two other diabetes groups supports this notion, and indicates that general practitioners are more likely to screen for diabetes in morbidly obese than in overweight individuals. Indeed, our findings support the “prevention paradox,” proposed by Geoffrey Rose more than 30 years ago, in which he stated that “a large number of people exposed to a low risk is likely to produce more cases than a small number of people exposed to a high risk” [21]. In the context of diabetes prevention, it may therefore not be optimal to focus only on promoting weight loss in the most obese individuals, but also aiming at preventing small weight gains in the entire population (i.e., shifting the entire BMI distribution to the left). This will only give a small benefit to each individual, but may prove effective at the population level in terms of preventing diabetes and CVD events in the future. The progressive weight gainers exhibited a pattern of obesity development with two separate phases of weight gain before type 2 diabetes diagnosis. Individuals with this pattern had an exponential increase in both insulin levels and beta cell function before the diagnosis of diabetes. Of interest, we found increased IL-1Ra concentrations towards diabetes diagnosis in the group of progressive weight gainers, but not in the persistently obese group. A previous analysis of the Whitehall II study found that IL-1Ra concentrations accelerated 6 years before diagnosis of diabetes, and differences between individuals who developed diabetes and those remaining diabetes-free were attenuated by adjustment for BMI or waist circumference [5]. Comparing IL-1RA trajectories with concurrent obesity development in our study, it seems that low-grade inflammation may predominantly be determined by changes in body weight and to a lesser extent by changes in glucose metabolism or actual level of obesity. Whether this applies to the general population needs further investigation.

Despite an increase in both obesity and systolic blood pressure over time, the calculated 10-year CVD risk from the Framingham CVD risk score was lower among the progressive weight gainers than among the other two groups at time of diabetes diagnosis, whereas the persistently obese and stable overweight groups had similar levels of estimated CVD risk despite different trajectories of CVD risk factors. These findings question the validity of calculated scores for disease risk in a population with heterogeneous disease development. Thus, future risk prediction models should aim to include knowledge about the heterogeneity of disease development instead of assuming a one-size-fits-all model.

The group of persistently obese individuals was on average characterised by class II obesity (35–40 kg/m^2^) [22] already 18 years before they were diagnosed with type 2 diabetes. Towards diabetes diagnosis this group experienced a pattern of beta cell compensation followed by loss of beta cell function, whereas insulin resistance only increased slightly. This pattern of beta cell dysfunction has for many years been thought to be characteristic for type 2 diabetes development [23]. However, this suggestion was originally based on a cross-sectional study of 82 individuals with long-standing (∼8–32 years) obesity and ∼8 years duration of type 2 diabetes [24], which questions the generalizability to leaner individuals with newly diagnosed diabetes. Thus, as evident from our data, the natural history of beta cell dysfunction in type 2 diabetes seems to be even more complex than previously thought and indeed not similar in all people developing type 2 diabetes [25].

A major strength of the Whitehall II study is its 18-year follow-up period and the detailed phenotypic characterisation of the study participants, which enabled us to relate trajectories of obesity development to trajectories of clinically relevant measures of metabolism and cardiovascular risk. Most previous studies examining BMI development over time have assumed that there is an average pattern of obesity development over time applying to the whole population [11],[26]–[28]. Another often used approach is to classify BMI into pre-defined categories, as currently debated in relation to mortality [29]. Limitations of this approach are that available information is not utilised optimally [30], and it may cause misclassification of individuals, especially near the cut-points for classification [31]. Moreover, the use of such more or less arbitrarily set cut-points keeps research locked in pre-defined concepts and thought patterns, which may prevent research moving forward. Instead of studying changes in predefined BMI categories, we chose to define subgroups of individuals by latent class trajectory analysis. This type of statistical method is useful to explore heterogeneous growth patterns that would not be identified by use of conventional methods. Indeed, latent class trajectory analysis is more flexible, because it models group-specific average patterns of obesity development. One disadvantage of latent class analysis is, however, that it often results in very different sizes of subgroups [11],[32], potentially limiting statistical power as well as interpretation and generalizability of the results. Another factor limiting the generalizability of our results is that this analysis was performed only in white participants. The reason for studying obesity development in an ethnically homogenous population was to avoid identifying differences in BMI patterns mainly attributed to ethnic differences. Therefore, these analyses should be confirmed in other study populations. In conclusion, latent class trajectory analysis identified three distinct patterns of obesity development leading to type 2 diabetes. The accompanying trajectories of insulin resistance and other cardiometabolic risk factors differed between these groups. In general, the majority of individuals developing type 2 diabetes were rather weight stable during follow-up with a slightly higher average BMI than the diabetes-free population, suggesting that strategies focusing on small weight reductions for the entire population may be more beneficial than predominantly focusing on weight loss for high-risk individuals.

## Supporting Information

Figure S1 **Trajectories for a hypothetical female of 50 years at time 0 of body mass index from 18 years before time of diagnosis/last examination.** Solid lines indicate estimated trajectories for each group and dashed lines are 95% confidence limits. Black bars at the bottom indicate the relative data distribution over the follow-up period. Light blue, stable overweight; dark blue, progressive weight gain; red, persistently obese; grey, diabetes-free population.
(TIF)

## Abbreviations

BMI: body mass index
CVD: cardiovascular disease
HDL: high-density lipoprotein
HOMA: homeostasis model assessment
IL-1Ra: interleukin 1 receptor antagonist
LDL: low-density lipoprotein
OGTT: oral glucose tolerance test
