# Supplementary figures and images for: Patterns of Obesity Development before the Diagnosis of Type 2 Diabetes: The Whitehall II Cohort Study
Source: PLoS Med. 2014 Feb 11;11(2):e1001602. doi: 10.1371/journal.pmed.1001602 (PMC3921118; doi:10.1371/journal.pmed.1001602)

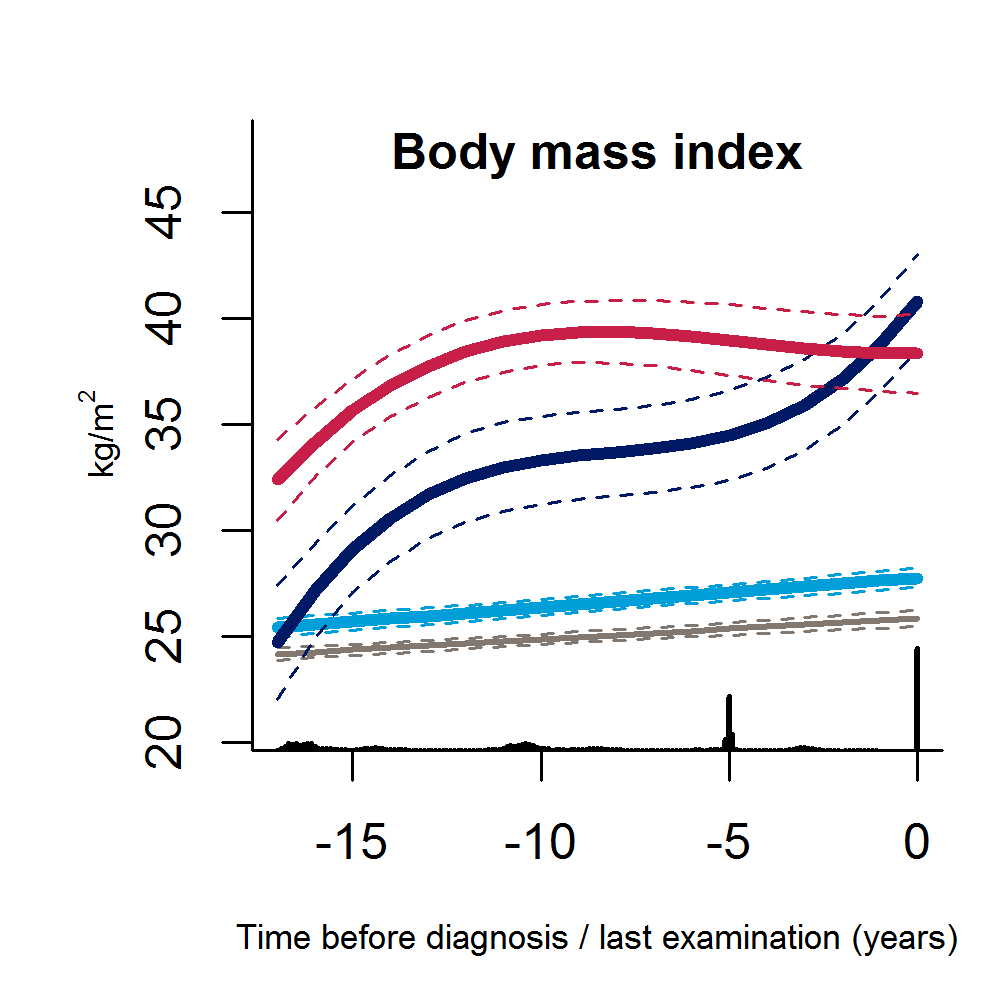

Supplement: Figure S1 — Trajectories for a hypothetical female of 50 years at time 0 of body mass index from 18 years before time of diagnosis/last examination. Solid lines indicate estimated trajectories for each group and dashed lines are 95% confidence limits. Black bars at the bottom indicate the relative data distribution over the follow-up period. Light blue, stable overweight; dark blue, progressive weight gain; red, persistently obese; grey, diabetes-free population. (TIF) [file pmed.1001602.s001.tif]
